# Framing Surgical Decisions in Elderly Patients: Minimally Invasive Partial Versus Radical Nephrectomy for Stage I Renal Cell Carcinoma at Mid-Term Follow-Up

**DOI:** 10.3390/jcm14186634

**Published:** 2025-09-20

**Authors:** Umberto Anceschi, Antonio Tufano, Rocco Simone Flammia, Eugenio Bologna, Riccardo Mastroianni, Leslie Claire Licari, Aldo Brassetti, Maria Consiglia Ferriero, Alfredo Maria Bove, Gabriele Tuderti, Simone D’Annunzio, Maddalena Iori, Silvia Cartolano, Marco Pula, Costantino Leonardo, Giuseppe Simone

**Affiliations:** 1 Department of Urology, IRCCS “Regina Elena” National Cancer Institute, 00144 Rome, Italy; 2 Department of Urology, San Carlo di Nancy Hospital GVM Care and Research, 00165 Rome, Italy

**Keywords:** renal cell carcinoma, RCC, minimally invasive partial nephrectomy, radical nephrectomy, elderly, octagenarians

## Abstract

**Background/Objectives**: The optimal surgical approach for stage I renal cell carcinoma (RCC) in ultra-octogenarians remains debated, especially when balancing oncologic control, renal preservation, and procedural safety. While ablative techniques and active surveillance are often favored in frail patients, robust comparative evidence supporting nephron-sparing surgery in this age group is limited. **Methods**: We retrospectively reviewed consecutive patients aged ≥80 years who underwent minimally invasive surgery for cT1 clear cell RCC at a high-volume tertiary-care center between July 2001 and August 2025. Patients were stratified into two cohorts: minimally invasive partial nephrectomy (MIPN, *n* = 51) and radical nephrectomy (MIRN, n = 26). All MIPNs were performed using an off-clamp approach. Baseline, perioperative, functional, and oncologic outcomes were compared. Kaplan–Meier analysis estimated overall survival (OS), cancer-specific survival (CSS), and progression to significant chronic kidney disease (sCKD, defined as CKD stage ≥ 3b). **Results**: Groups were comparable in age, comorbidities, and ASA score. MIRN patients exhibited higher tumor complexity (RENAL score: 9 vs. 7, *p* = 0.01) and a greater proportion of pT1b lesions (77% vs. 37.3%, *p* = 0.01). Perioperative transfusions occurred exclusively in the MIRN group (*p* = 0.01), whereas complication rates were low and similar between groups. MIPN was associated with significantly higher eGFR at follow-up (48 vs. 30.9 mL/min/1.73 m^2^, *p* = 0.01) and a delayed progression to sCKD (*p* = 0.01), with no differences in OS or CSS at a median follow-up of 30.5 months. **Conclusions**: In this real-world series of ultra-octogenarians with cT1 clear cell RCC, off-clamp minimally invasive partial nephrectomy ensured superior renal function preservation and delayed progression to sCKD, without compromising oncologic control at mid-term follow-up. Beyond statistical outcomes, these results underscore the importance of tailoring surgical strategies to protect long-term functional autonomy and preserve physiological resilience in elderly patients.

## 1. Introduction

The optimal management of stage I renal cell carcinoma (RCC) in ultra-octogenarians remains a matter of clinical debate [1,2]. In this fragile and often underrepresented population, treatment decisions must carefully balance oncologic control, renal function preservation, and procedural safety [3,4]. For small renal masses, active surveillance has emerged as a viable strategy in elderly patients, particularly when comorbidities or limited life expectancy reduce the value of aggressive intervention [5,6,7]. Ablative approaches such as cryoablation and radiofrequency ablation also represent alternatives, though concerns persist regarding long-term efficacy and consistent functional outcomes [8,9,10].

Minimally invasive partial nephrectomy (MIPN) has progressively gained traction in selected elderly patients, supported by improvements in surgical technique and growing awareness of the benefits of nephron preservation [4,11]. Nonetheless, comparative evidence between MIPN and minimally invasive radical nephrectomy (MIRN) in patients aged ≥ 80 years remains limited—particularly regarding mid-term outcomes in terms of survival and renal function [12,13]. Notably, the adoption of off-clamp enucleative techniques has opened new perspectives for reducing ischemic stress in patients with fragile renal reserve, a factor of particular importance in the elderly [14,15,16]. In this context, we present a single-centre, real-world study from a tertiary referral institution comparing MIPN and MIRN in ultra-octogenarians with cT1 RCC, focusing on perioperative outcomes, renal function dynamics, and survival—at a mid-term follow-up.

## 2. Materials and Methods

### 2.1. Study Design and Population

This is a retrospective, single-center cohort study conducted at a high-volume tertiary-care referral institution (IRCCS Regina Elena National Cancer Institute, Rome, Italy), including all consecutive patients aged ≥ 80 years who underwent minimally invasive surgery for clinical stage T1 RCC between July 2001 and August 2025. All procedures were performed by experienced urologic oncologic surgeons. Institutional review board approval was obtained, and the study was registered under protocol code RCC80-IRCCS2025.

Patients were considered eligible if they were ≥80 years at the time of surgery, had a preoperative radiologic diagnosis of clinical stage T1 RCC, and had complete preoperative and postoperative renal function data available. Only cases with final histologic confirmation of clear cell RCC (ccRCC) were retained for analysis. No patient underwent preoperative renal biopsy.

To ensure homogeneity of oncologic risk and baseline functional reserve, we excluded patients with multifocal tumors, metastatic disease at presentation, benign or non-RCC malignancies at final pathology, congenital renal anomalies (including horseshoe kidney), and preoperative estimated glomerular filtration rate (eGFR) values < 30 mL/min/1.73 m^2^. Patients who had previously undergone ablative treatments—such as cryoablation or radiofrequency ablation—were also excluded, as these are not routinely performed at our institution. All minimally invasive partial nephrectomies (MIPN) were performed using a standardized off-clamp approach.

### 2.2. Study Groups and Surgical Approach

Patients were stratified into two groups based on the surgical modality received. Group A included patients who underwent minimally invasive partial nephrectomy (MIPN; n = 51), performed via laparoscopic or robot-assisted techniques. Group B comprised patients who received minimally invasive radical nephrectomy (MIRN; n = 26), also through laparoscopic or robotic access. The choice between minimally invasive partial nephrectomy and radical nephrectomy was determined according to predefined clinical parameters, including tumor size, RENAL complexity score, and baseline renal function, with final allocation left to the judgment of the operating surgeon.

### 2.3. Variables and Outcomes

Baseline variables collected included age (years), body mass index (BMI, kg/m^2^), American Society of Anesthesiologists (ASA) score (I–II vs. III–IV), tumor laterality (right vs. left), history of hypertension (yes/no), diabetes mellitus (yes/no), tumor size (mm), R.E.N.A.L. nephrometry score (reported as median with interquartile range [IQR]), and solitary kidney status (yes/no).

Perioperative parameters included surgical approach (robotic vs. laparoscopic), reconstructive technique in the MIPN group (sutureless vs. renorrhaphy), preoperative hemoglobin (g/dL), preoperative eGFR (mL/min/1.73 m^2^, calculated using the CKD-EPI formula), length of hospital stay (LOS, days), perioperative complications (graded according to the Clavien–Dindo classification, stratified as grade I–II vs. grade III–V), and requirement for perioperative blood transfusions (yes/no).

Pathological data comprised pathological T stage (pT1a vs. pT1b), surgical margin status (positive vs. negative), and histologic subtype (clear cell RCC only). Functional outcomes included postoperative eGFR at last follow-up (mL/min/1.73 m^2^), occurrence of newly diagnosed end-stage renal disease (ESRD, defined as CKD stage 4 or 5 or need for chronic dialysis), and duration of follow-up (months, reported as median with IQR).

### 2.4. Endpoints

The primary endpoint of the study was to compare baseline characteristics, perioperative outcomes, and renal functional results between patients undergoing MIPN and those treated with MIRN.

The secondary endpoint was to assess and compare oncologic and functional survival outcomes between the two cohorts using Kaplan–Meier analysis. These included overall survival (OS), cancer-specific survival (CSS), and freedom from progression to significant chronic kidney disease (sCKD), defined as CKD stage ≥ 3b.

### 2.5. Statistical Analysis

Continuous variables were reported as medians and interquartile ranges (IQR) and compared using the Mann–Whitney U test. Categorical variables were expressed as absolute frequencies and percentages and analyzed using Pearson’s χ^2^ test or Fisher’s exact test, as appropriate. Kaplan–Meier curves were constructed to estimate OS, CSS, and sCKD-free survival; group comparisons were performed using the log-rank test. All statistical tests were two-sided, and a *p*-value <0.05 was considered statistically significant. Statistical analyses were performed using IBM SPSS Statistics, version 28.0 (Armonk, NY, USA).

## 3. Results

Baseline characteristics revealed substantial clinical comparability across groups, with no significant differences observed in age, body mass index (BMI), ASA score, or comorbidities such as hypertension and diabetes (each *p* > 0.4; Table 1). However, patients in the MIRN group presented with a significantly higher proportion of left-sided tumors (73.1% vs. 45.1%, *p* = 0.02) and more complex renal masses, as reflected by elevated RENAL scores (median 9 [IQR 8–9] vs. 7 [IQR 6–9], *p* = 0.01). Median tumor size was also larger in the MIRN cohort (50 mm [IQR 42.2–60] vs. 35 mm [IQR 25–45], *p* = 0.25). Regarding surgical approach, robotic techniques were more frequently employed across both groups, with no significant difference in distribution (*p* = 0.34). All partial nephrectomies were performed using an off-clamp technique, and 39.2% of these cases were completed without renorrhaphy.

Perioperative outcomes were favorable in both groups. Length of hospital stay (LOS) was comparable (2 days [IQR 2–5] in MIPN vs. 3.5 days [IQR 2–6] in MIRN, *p* = 0.45), and no significant differences were observed in overall complication rates (each n = 3, *p* = 0.54). Complications were uniformly low-grade (Clavien I–II), including two cases of ileus and one febrile episode in the MIPN cohort, and three cases of postoperative anemia requiring transfusion in the MIRN group. No high-grade complications (Clavien III–V) or hospital readmissions at 30 or 90 days were recorded in either group. Perioperative transfusions were required exclusively in the MIRN group (0% vs. 11.5%, *p* = 0.01). Pathologic evaluation showed a significantly higher rate of pT1b tumors in the MIRN series (77% vs. 37.3%, *p* = 0.01), while surgical margins were negative in all cases.

At a median follow-up of 30.5 months (IQR 10–57.5), functional assessment revealed a significantly better preservation of renal function in the MIPN group, with higher median eGFR values (48 vs. 30.9 mL/min/1.73 m^2^, *p* = 0.01) and a similar incidence of newly onset end-stage renal disease (ESRD: 1 vs. 2 cases, *p* = 0.14; Table 1).

**Figure 1:**
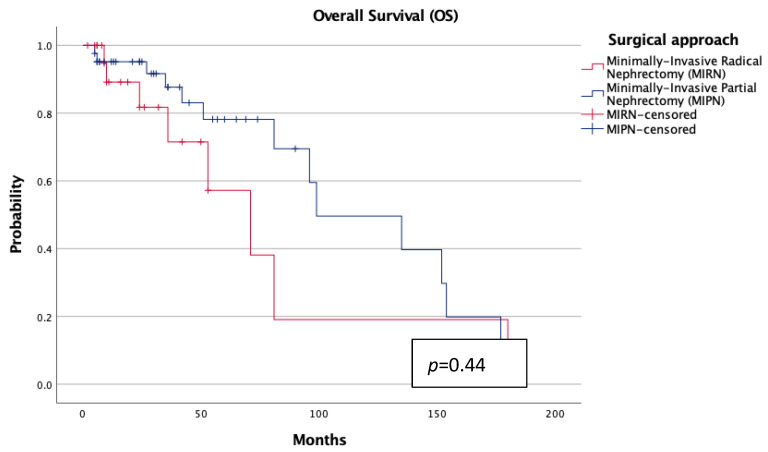
Overall survival according to surgical approach.

**Figure 2:**
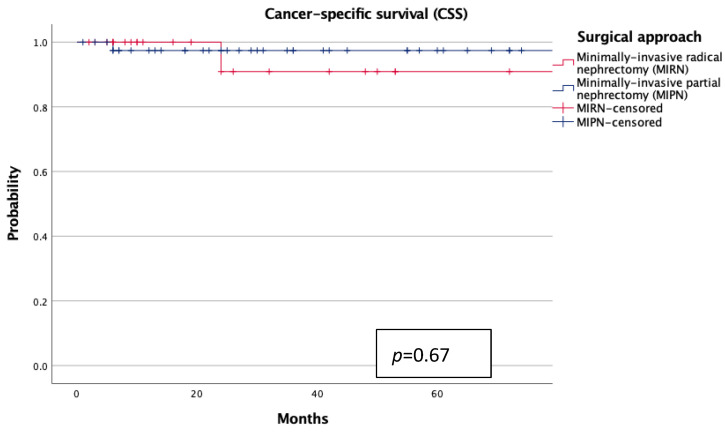
Cancer-specific survival (CSS) according to surgical approach.

**Figure 3:**
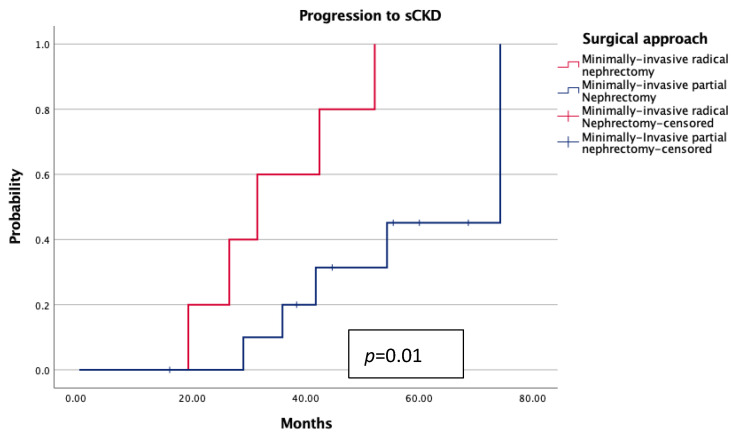
Probability of progression to sCKD according to surgical approach.

Kaplan–Meier analyses demonstrated no statistically significant differences in overall survival (OS) or cancer-specific survival (CSS) between groups (each *p* > 0.7; Figure 1 and Figure 2). However, patients treated with MIRN exhibited a significantly earlier progression to chronic kidney disease stage ≥3b (sCKD) (*p* = 0.01; Figure 3).

## 4. Discussion

The optimal management of localized renal cell carcinoma (RCC) in octogenarians remains a matter of debate, particularly in light of evolving surgical paradigms, competing oncologic and functional priorities, and the heterogeneous physiological status of elderly patients [3,4,17,18]. Within this complex scenario, our study provides a focused comparative analysis of minimally invasive partial (MIPN) versus radical nephrectomy (MIRN) in patients aged ≥80 years with clinical stage T1 RCC, aiming to inform surgical decision-making in this age group, where evidence is scarce and often extrapolated from younger populations [19].

Despite a comparable distribution of key baseline characteristics—such as age, comorbidities, and ASA score—we observed a significantly higher prevalence of left-sided tumors and greater tumor complexity, as assessed by RENAL score, in the MIRN cohort (each *p* < 0.03). These findings suggest a potential influence of tumor anatomy on the surgical indication, favoring MIRN in more challenging cases. The higher RENAL complexity may also account for the greater rate of pT1b tumors and the need for radical extirpation over nephron-sparing approaches [15,20]. Notably, perioperative transfusions were observed exclusively in the MIRN group (*p* = 0.01), likely reflecting both higher tumor burden and the increased vascular manipulation intrinsic to radical surgery.

From a functional standpoint, MIPN was associated with a significantly higher median eGFR at follow-up (*p* = 0.01), despite similar preoperative values. This suggests a true nephron-sparing benefit, particularly relevant in elderly patients with reduced renal reserve. Although the incidence of ESRD was low and not statistically different, Kaplan–Meier analysis revealed a significantly earlier progression to sCKD in the MIRN group (*p* = 0.01), reinforcing the concept that radical nephrectomy, even in technically successful procedures, may accelerate renal decline over time. The implications of this functional deterioration—though not immediately fatal—should not be underestimated, as it may increase frailty, cardiovascular vulnerability, therapeutic burden, and ultimately compromise autonomy and global health status in elderly individuals. Notably, oncologic outcomes were comparable between the two cohorts, with no statistically significant differences in overall or cancer-specific survival at a mid-term follow-up. This finding aligns with recent data supporting the non-inferiority of MIPN in appropriately selected elderly patients and questions the routine adoption of radical surgery when a nephron-sparing approach is technically feasible [4,21]. These observations are also consistent with larger series conducted in younger or mixed-age populations, where partial nephrectomy has repeatedly shown non-inferior CSS and OS compared with radical nephrectomy. Taken together, this body of evidence reinforces the concept that nephron-sparing surgery ensures oncologic safety across different age groups, while our study specifically extends this paradigm to ultra-octogenarians, an underrepresented yet clinically relevant cohort.

Nonetheless, our study is not devoid of limitations. First and foremost, the retrospective and non-randomized design introduces multiple biases—chiefly selection bias—influencing the allocation to surgical approach based on tumor complexity and surgeon discretion. The lack of propensity score matching, though acknowledged, is a methodological limitation that may amplify baseline imbalances. In principle, a propensity score adjustment would have strengthened comparability between groups. However, given the restricted cohort size and the uneven distribution of key clinical variables, its application would have inevitably reduced the analyzable sample to an unrealistic number of patients, thereby undermining statistical reliability rather than enhancing it. For this reason, we deliberately refrained from forcing an underpowered adjustment, opting instead to provide a transparent appraisal of baseline imbalances and to acknowledge this limitation as intrinsic to the present single-center design. Moreover, residual confounding by indication is plausible. Allocation to MIRN tended to occur in anatomically more complex or larger tumors, whereas comprehensive geriatric or frailty indices were not systematically collected across the accrual period. As a result, unmeasured differences in physiological reserve and anatomy may have influenced both treatment selection and outcomes, beyond the covariates captured in our dataset. Attrition bias cannot be excluded, particularly given the variable length of follow-up across groups. Moreover, the small sample size, especially in the MIRN cohort, limits the power of subgroup analyses and the generalizability of our findings.

Importantly, our dataset did not include patients undergoing ablative treatments such as cryoablation or radiofrequency ablation, which are increasingly adopted in elderly patients with limited life expectancy or impaired performance status. While this enhances the internal consistency of our surgical cohort, it restricts external applicability and reflects institutional practice patterns rather than broader oncologic strategies. In addition, no preoperative renal biopsies were performed, raising the potential for overtreatment of benign or indolent lesions, although all included cases were pathologically confirmed as clear cell RCC. Furthermore, although functional outcomes were rigorously assessed via standardized eGFR measurements and survival analysis, no patient-reported outcomes or quality-of-life data were available. These parameters, though difficult to collect retrospectively, are highly relevant in geriatric populations and may influence treatment value more than traditional oncologic metrics. Finally, all procedures were performed in a high-volume tertiary-care referral center by experienced urologic oncologists. While this ensures surgical standardization and internal validity, it may not reflect the outcomes achievable in lower-volume or non-specialized settings, potentially limiting external validity. From a methodological perspective, we recognize that a robust propensity-based comparison can only be realistically achieved in larger, preferably multicenter, datasets. Such collaborations will be essential to validate the present findings and to better delineate the decision-making process guiding surgical choice in ultra-octogenarians, where individual surgeon discretion and anatomical complexity are often closely intertwined. Accordingly, our findings should be interpreted as real-world effectiveness signals rather than definitive causal estimates; they are hypothesis-generating and intended to inform adequately powered, multicenter prospective cohorts with standardized geriatric and morphometric covariates. Finally, the median follow-up of 30.5 months, although sufficient to assess perioperative and mid-term functional trends, is not adequate to draw conclusions on the long-term trajectories of renal decline, overall survival, or late competing risks in this frail population. Our results should therefore be interpreted as mid-term signals, with the understanding that extended follow-up and larger prospective cohorts will be essential to validate whether the functional advantages of MIPN translate into durable clinical benefits over time. Another limitation is the lack of systematically recorded operative time across the entire accrual period. Given the long study timeframe and the transition between laparoscopic and robotic platforms, operating times were not consistently documented in a standardized format. As a result, they could not be reliably analyzed, and we deliberately refrained from reporting incomplete data. Future studies should prospectively capture perioperative efficiency metrics, including operative time, to better characterize the overall procedural burden in elderly patients.

Despite these limitations, our study contributes robust mid-term evidence supporting the safety and functional superiority of MIPN in well-selected ultra-octogenarians. The uniform adoption of the off-clamp technique in the MIPN cohort, performed without hilar clamping or ischemia, may further enhance renal preservation and mitigate intraoperative stress, although this remains speculative without a direct clamping comparison. Looking ahead, ultra-octogenarians are increasingly becoming a standard population in contemporary urologic practice, rather than a marginal exception. As life expectancy continues to rise and healthcare systems adapt to the needs of older adults, this subgroup is expected to occupy a progressively central role in surgical decision-making. Therefore, refining surgical strategies, optimizing risk stratification, and tailoring treatment algorithms in this age segment will remain a critical and growing topic in the years to come.

## 5. Conclusions

In this single-center study, minimally invasive partial nephrectomy (MIPN) was associated with superior preservation of renal function and lower perioperative morbidity compared to radical nephrectomy (MIRN) in ultra-octogenarians with clinical stage T1 clear cell RCC, without compromising oncologic safety at mid-term follow-up. These findings suggest that, in appropriately selected elderly patients, nephron-sparing surgery may represent the preferred option whenever anatomically and technically feasible. As ultra-octogenarians become a growing part of daily urologic practice, individualized treatment planning should increasingly incorporate not only tumor control and functional outcomes, but also the broader implications for patients’ long-term autonomy and quality of life.

## Figures and Tables

**Table 1:** Baseline, perioperative, pathologic and functional outcomes.

Variable	MIPN (n = 51) Group A	MIRN (n = 26) Group B	p Value
Age (years, median, IQR)	81.9 (80.8–83.5)	83 (80.6–84.5)	0.23
BMI (Kg/m^2^, median, IQR)	26.4 (23.4–27.7)	25.3 (22.8–28.4)	0.81
ASA score (n,%) 1–2 3–4	29 (56.9%) 22 (43.1%)	17 (65.3%) 9 (34.7%)	0.82
Side (n,%) Right Left	28 (54.9%) 23 (45.1%)	7 (26.9%) 19 (73.1%)	**0.02**
Hypertension (n,%)	29 (56.8%)	14 (52%)	0.72
Diabetes (n,%)	7 (13.7%)	5 (19.2%)	0.46
Tumor size (mm, median, IQR)	35 (25–45)	50 (42.2–60)	0.25
RENAL score (median, IQR)	7 (6–9)	9 (8–9)	**0.01**
Solitary kidney (n,%)	3 (5.8%)	-	**-**
Surgical approach (n,%) Laparoscopic Robotic	16 (31.3%) 35 (68.7%)	11 (42.3%) 15 (57.7%)	0.34
Reconstructive technique (n,%) Sutureless Renorraphy	20 (39.2%) 31 (60.8%)	- -	-
Preoperative Hb (g/dL, median, IQR)	13.4 (12.3–14.4)	12.7 (12.2–14.3)	0.52
Preoperative eGFR (median, IQR)	61.5 (54.5–77–5)	54 (39.5–64.5)	0.62
LOS (days, median, IQR)	2 (2–5)	3.5 (2–6)	0.45
Perioperative Complications (n, detail, %) Clavien I–II Clavien III–V	3 (5.8%) 3 (2 Ileus, 1 Fever) -	3 (11.5%) 3 (Anemia)	0.54
Perioperative transfusions (n,%)	**-**	3 (11.5%)	**0.01**
30-days readmission (n,%)	**-**	**-**	**-**
90-days readmission (n,%)	**-**	**-**	**-**
pT stage (n,%) pT1a pT1b	32 (62.7%) 19 (37.3%)	6 (23%) 20 (77%)	**0.01**
Surgical margin status (n,%)	-	-	**-**
Median follow-up (months, median, IQR)	36 (13.7–70.2)	21.5 (9–48)	0.15
eGFR follow-up (median, IQR)	48 (38.8–64.2)	30.9 (27.2–39.6)	**0.01**
ESRD (n,%)	1	2	0.14

MIPN: Minimally invasive partial nephrectomy; MIRN: minimally invasive radical nephrectomy; IQR: interquartile ranges; BMI: body mass index; ASA: American Society of Anesthesiologists; eGFR: estimated glomerular filtration rate; Hb: Hemoglobin; LOS: Length of Stay; ESRD: End-stage renal disease.

## Data Availability

The data presented in this study are available on request from the corresponding author.

## Abbreviations

The following abbreviations are used in this manuscript:

RCC	renal cell carcinoma
MIPN	minimally invasive partial nephrectomy
MIRN	minimally invasive radical nephrectomy
ccRCC	clear cell RCC
eGFR	estimated glomerular filtration rate
BMI	body mass index
ASA	American Society of Anesthesiologists
LOS	length of hospital stay
IQR	interquartile ranges
sCKD	significant chronic kidney disease
ESRD	end-stage renal disease
OS	overall survival
CSS	cancer-specific survival
